# Plant, pigment, and bone processing in the Neolithic of northern Arabia–New evidence from Use-wear analysis of grinding tools at Jebel Oraf

**DOI:** 10.1371/journal.pone.0291085

**Published:** 2023-10-04

**Authors:** Giulio Lucarini, Maria Guagnin, Ceri Shipton, Anita Radini, Abdullah M. Alsharekh, Michael Petraglia

**Affiliations:** 1 Institute of Heritage Science, National Research Council of Italy, Rome, Italy; 2 Department of Asian, African, and Mediterranean Studies, University of Naples L’Orientale, Naples, Italy; 3 Department of Archaeology, Max Planck Institute of Geoanthropology, Jena, Germany; 4 Institute of Archaeology, University College London, London, United Kingdom; 5 School of Archaeology, University College Dublin, Dublin, Ireland; 6 Department of Archaeology, College of Tourism and Archaeology, King Saud University, Riyadh, Saudi Arabia; 7 Australian Research Centre for Human Evolution, Griffith University, Nathan, Australia; 8 School of Social Science, University of Queensland, St Lucia, Australia; 9 Human Origins Program, National Museum of Natural History, Smithsonian Institution, Washington, D.C., United States of America; New York University, UNITED STATES

## Abstract

Archaeological sites with surface hearths are a ubiquitous feature across the arid zones of the Arabian interior. At Jebel Oraf, in the Jubbah basin of the Nefud Desert of northern Arabia, numerous grinding stone fragments were found in association with hearths, though the original purpose of these stones was unclear owing to the poor preservation of faunal and botanic remains. Here we describe results from use-wear analysis on five grinding tools at Jebel Oraf, demonstrating that such artefacts were used during the Neolithic for plant processing, bone processing, and pigment production. Grinding stones were often broken up after initial use and fragments were subsequently re-used for alternative purposes, before finally being placed on hearths or discarded. More specifically, plants were ground or prepared and possibly cooked in the hearths, and bones were processed as well. The analyses also highlight the importance of pigment processing at Neolithic sites and provide a link to painted rock art. The frequent use of pigment in the archaeological record suggests that pigment was widely used, and that Neolithic painted art may have been more common than the surviving images suggest.

## Introduction

The Neolithic in Arabia has been characterised by the introduction of domesticated livestock and a transition from hunting to a mixed economy of mobile pastoralism alongside hunting [1]. The marginal environment of northern Arabia seems to have led to a more selective adoption of characteristics that are typically associated with the Neolithic in the Levant. Sedentism and agriculture have not been documented until the Bronze Age, when the onset of arid conditions is associated with more intensive occupation of oases, and intensification of plant cultivation, sustained through sophisticated water management systems [2]. In Tayma, an oasis in the northwest of the Arabian Peninsula, oasis cultivation of grapevine and fig is attested from 4600 BC [3]. Similar shifts in subsistence and mobility patterns are also known from the Sahara where mobile pastoral lifeways gave way to oasis settlements following the end of the Holocene humid period, and culminated in the rise of major polities such as the Garamantian Kingdom of southern Libya (1000 BC–AD 500) [e.g. 4, 5]. The Mid- Holocene herders of Arabia lacked characteristics such as sedentism and pottery production, but Levantine traits in the lithic industries, particularly distinctive types of pressure-flaked bifacial arrowheads, indicate repeated contact evident over millennia [1]. The substantial socio-economic changes heralded by the introduction of livestock herding and ownership are commonly associated with the Neolithic [6]. However, the extent of this economic shift is still uncertain in northern Arabia. Cattle feature prominently in the rock art of this period, yet faunal assemblages recovered from Neolithic sites are often dominated by wild species, for example at Jebel Oraf, and at the Camel Site [1, 7]; while faunal remains from ritual contexts often consist of a mixture of wild and domesticated species [8, 9]. It is also not known to what extent the exploitation of wild or domesticated plants was part of the subsistence economy. At Tayma, *Cerealia* pollen recovered from lake deposits have been argued to provide evidence for cereal cultivation in the Neolithic [10], however, no plant remains have yet been recovered from excavated Neolithic sites in the region.

One of the main challenges hampering a reconstruction of Neolithic subsistence strategies and other activities is the poor preservation of organic materials in the arid environments of northern Arabia. Ritual activities are visible in the form of large stone structures [e.g. 11], and rock art sites [12], while mobility patterns can be inferred from occupation sites [1, 13]. Hunting is evident in the sparse faunal record (see above), and also in the presence of large hunting structures, known as kites [14]. To date, there is only sporadic evidence of pigment use in northern Arabia. Groucutt and colleagues [11] report a stone that was painted with a red geometric pattern and had been integrated into the walls of a mustatil, suggesting that pigment may have played a role in the activities that took place in and around these monumental structures. In the absence of organic remains, processing of meat and bones can only be documented through the characteristics and use-wear of stone tools, which are abundant in the archaeological record [e.g. 15]. The use of large-scale traps, which has recently been documented on the north-eastern edge of the Nefud Desert [14, see also 16] certainly suggests large quantities of meat may have required processing and preservation to facilitate storage or trade of surplus.

**Fig 1:**
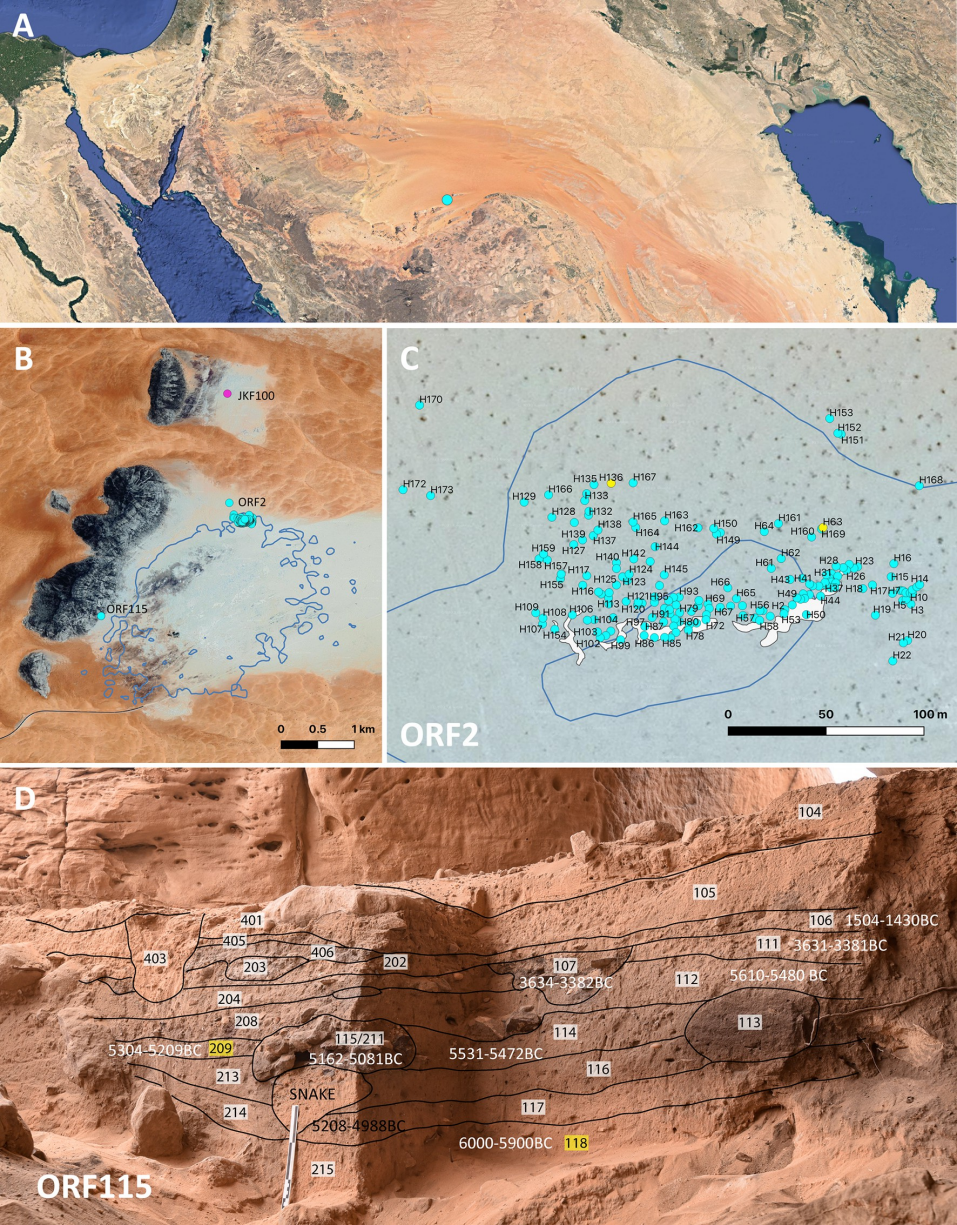
Map showing the location of the Jebel Oraf sites. A: Google Earth satellite image showing the location of Jebel Oraf in northern Arabia. B: Jebel Oraf palaeolake basin showing the location of sites mentioned in the text. Lake extent is modelled based on a high-stand dated to around 5300BC [17] C: Location of hearths at ORF2, hearths with grinding tools selected for use-wear analysis are marked in yellow. On the southern edge hearths are placed on top of grey lake marl deposits. Blue lines indicate a lake high stand [reported in 1, 17]. D: Stratigraphy and radiocarbon ages at ORF115 [reported in 1], contexts with grinding tools selected for use-wear analysis are marked in yellow. Radiocarbon age in black may have been moved/ contaminated by a snake hole above.

Fieldwork in the Jebel Oraf palaeolake basin, in the southern Nefud Desert of Saudi Arabia, has identified a Neolithic landscape around a palaeolake [17], with a grinding stone manufacturing site (JKF100) identified in the adjacent Jebel Katefeh basin (Fig 1). Excavations of a rockshelter (ORF115) and an open-air site (ORF2) showed repeated but short-lived occupations throughout the mid to late Holocene. Radiocarbon ages indicate a peak in occupation during the sixth and early fifth millennium BC, followed by more sporadic use of the site until the recent past [1]. The sites are characterised by small hearths, formed by making small depressions in the sand, around 50 cm in diameter and 10 to 20 cm deep. Most of the identified hearths were ephemeral and were likely in use for a matter of hours, rather than days, though a few more elaborate ones are lined with stones. These hearths were typically covered in small stones and grinding stone fragments, with the latter sometimes showing signs of having been broken intentionally prior to placement on the hearth. Grinding tools were numerous at the site, with 154 recovered from ORF2, and a further 11 from the Neolithic layers at ORF115. These tools were unevenly distributed, with one hearth yielding 45 pieces, including refit sets with up to 12 individual pieces. The grinding stones appear to have been used to cover the hearths–possibly to contain the fire or for use in cooking–but their original use remained unknown. The sheer number of grinding tool fragments at ORF2 and ORF115, and their deliberate breaking and placement on hearths, make them a key feature of the sites. Here we present detailed results of use-wear and micro-residue analysis of five grinding tools recovered from Jebel Oraf, that give some insight into the production, use, and reworking of these artefacts and allow a reconstruction of some of the activities that took place at these Neolithic sites.

## Background

**Fig 2:**
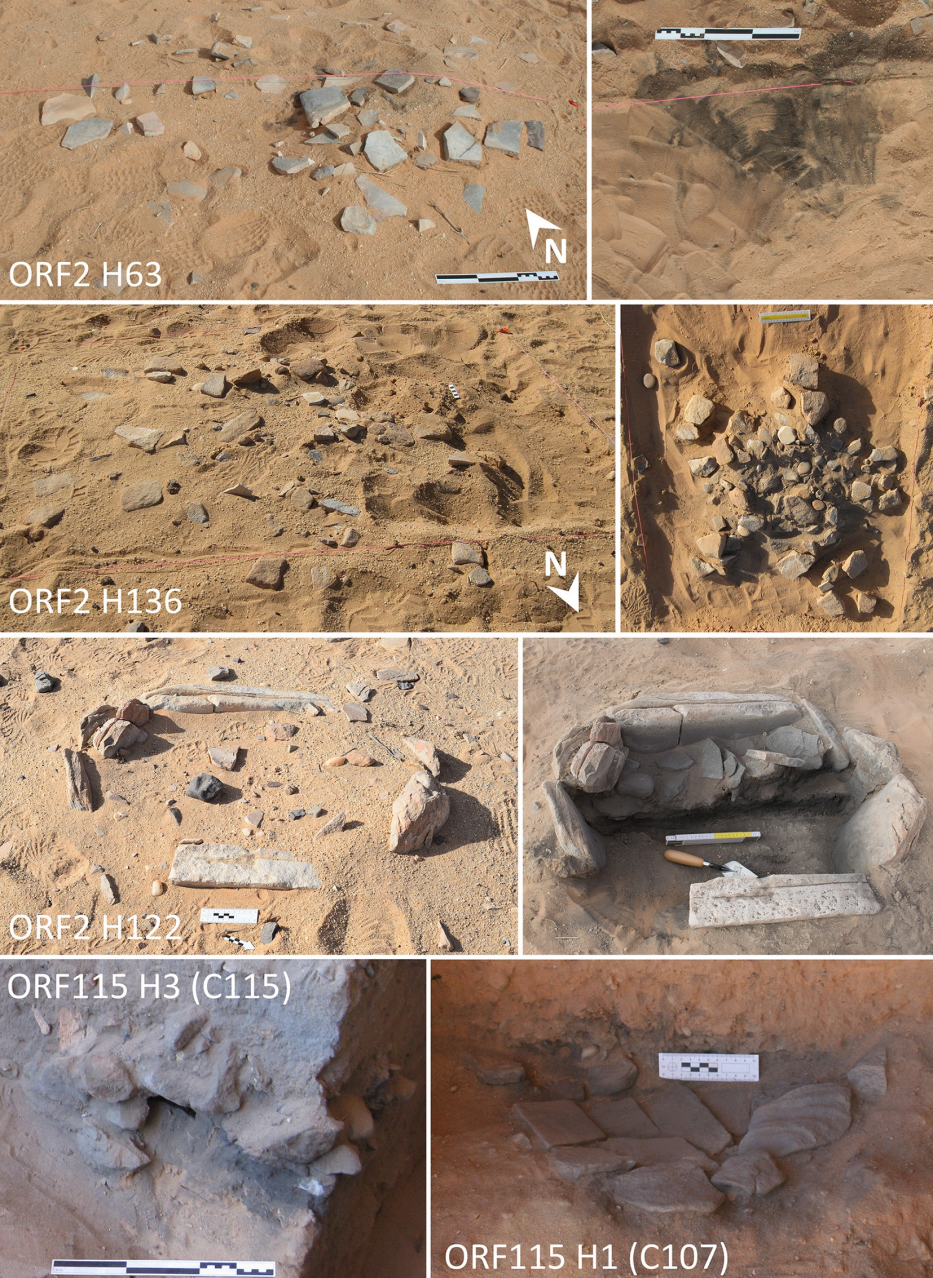
Hearths from ORF2 and ORF115. ORF2 hearths (top 3) are near the surface and are shown before (left) and after (right) excavation. Tool 4 was found in hearth 136, and tool 5 in hearth 63. Hearths at ORF115 were embedded in a stratified deposit visible on the edge of a looting hole and subsequently excavated.

The sites of ORF2 and ORF115 are located on the edge of a shallow palaeolake basin (Fig 1). ORF2 is situated on a grey lake marl deposit, at the base of a sand dune and would have been close to the edge of the water. A total of 170 hearths have been documented (Fig 1C), with most still visible on the surface in the shape of small clusters of stones that were placed on top of ashy deposits. Hearths are generally found just below the modern surface, although stones surrounding or covering the hearths are visible on the surface (Fig 2). The palaeolake appears to have filled towards the end of the Holocene humid period. A number of high lake stands were dated to the late 6^th^ millennium BC and shown to have destroyed earlier hearths at the site, causing ash, bone fragments, and flaked stone to become mixed with and embedded into the lake marl [17]. Thus far, 17 of the hearths have been excavated at ORF2; excavations have shown that there is limited stratigraphy at the site, beyond the placement of hearths on lake sediments, which are themselves embedded with the remains of earlier hearths.

**Fig 3:**
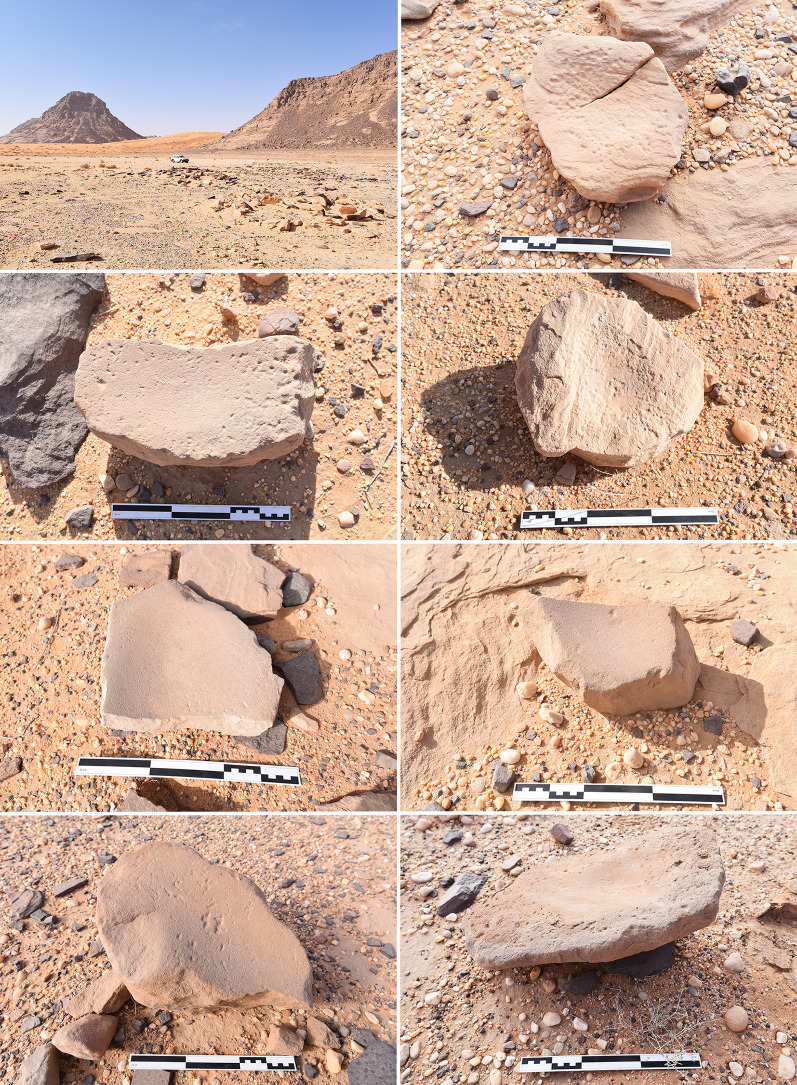
Site JKF100. View across the site (top left) and a selection of grinding stones in varying degrees of preparation through pecking and grinding found at the site. Scale is 30 cm long. Note that Jebel Oraf is visible in the background on the left of the top left image.

ORF115 is a stratified site inside a cluster of boulders that has formed a small shelter, located at the base of Jebel Oraf, a short distance from the palaeolake (Fig 1D). The lower half of the sequence dates to the sixth millennium BC, with later use of the shelter in the Bronze and Iron Ages [1] Unlike ORF2, the grinding tools at ORF115 that were selected for analysis were not directly associated with (surviving) hearths, however as the site had been looted their position may have related to deposits that were lost [1]. Jebel Katefeh (JKF100), just 1 km to the north of ORF2, appears to have been a grinding stone manufacturing area (Fig 3). Several dozen grinding tools are still visible on the surface, clustered over an area that is ca 30m in diameter. At least three cairns were subsequently built next to the site and can be dated to the later Holocene based on the absence of rock varnish. These cairns appear to have re-used some of the grinding tools in the construction process. The paucity of other lithics at the site suggests this was primarily a grinding stone production site, testifying to the local importance of this technology. It is possible that some of the grinding tools recovered in the Jebel Oraf basin came from this site.

Faunal remains recovered from ORF2 and ORF115 suggest a diverse subsistence strategy with the hunting of gazelle, oryx, and possibly ibex and the collection of ostrich eggs alongside the hunting or trapping of small birds. Fragments of *Bos* and *capra* may reflect herded livestock [1]. This picture of a mixed hunter/herder subsistence is also echoed at hearth sites recorded from the western Nefud Desert [13]. At Jebel Oraf, a high proportion of burnt bone, and an absence of gnawing suggest that the faunal remains are associated with the actual use of the hearths and the discarded bones became incorporated into the burning events [1]. This raises the question whether the grinding stone fragments, several of which also show evidence of burning, were used for cooking or drying meat.

Contemporary sites in the Levant are characterised by a complex subsistence strategy that may also have incorporated opportunistic agriculture [see for example: 18, 19]. It is therefore possible that grain cultivation, or at least grain processing, played a role in subsistence at Jebel Oraf. Evidence from the eastern and central Sahara show the important role played by wild plants within the broad-spectrum economy of Mid-Holocene desert dwellers [20–23]; which also included cattle and caprine herding, hunting of small and medium size mammals, and ostrich exploitation, mainly for eggs and plumage [24, 25]. Plant species, especially the wild grasses in the eastern Sahara, were largely processed with grinding tools [15, 26]. Unfortunately, due to poor preservation, macro-botanical remains were not recovered at Jebel Oraf, despite a dedicated nested sieving sampling strategy. Preservation of phytoliths was also very poor and processing was abandoned due to very low quantities of specimens [1].

In total, 19 top active stones were found (12 at ORF2 and 7 at ORF115), indicating that some grinding activity took place on site prior to the breaking and discarding of the bottom passive grinding stone elements. As the majority of these items were found on the surface, only a few of them were suitable for micro-residue analysis, however, the assemblage was suitable for use-wear analysis, which can provide important insights into their biography, including the materials they came into contact with during use.

The boulders forming the shelter of ORF115, as well as numerous other boulders in the vicinity, are densely covered in engravings. Several quartz and quartzite pebbles recovered from ORF2 showed extensive hammering traces that are consistent with their use as pecking stones to create petroglyphs [1]. While most of the Neolithic rock art of northern Arabia is engraved, the rock art at the Jubbah Oasis also includes a small number of painted panels [12]. It is therefore also possible that grinding tools were used for pigment processing.

## Materials

The grinding tool assemblage from ORF2 includes 150 bottom stones and 12 top active stones (the latter including three specimens from site reconnaissance in 2015 not reported in [1]). All except six grinding tools from ORF2 were manufactured using the local tabular quartzitic sandstone basement rock, with evidence for grinding stone production detected at the site of JKF100 in the basin in front of neighbouring Jebel Katefeh (Fig 3). The grinding stones at JKF100 are all quite thick (Fig 3) in comparison to those from ORF2 (where mean bottom stone thickness is just 25 mm), and it may be that thinner pieces were transported from JKF100 to ORF2, or there was an as yet unidentified production locality in the Oraf basin where the ORF2 grinding stones were made.

**Fig 4:**
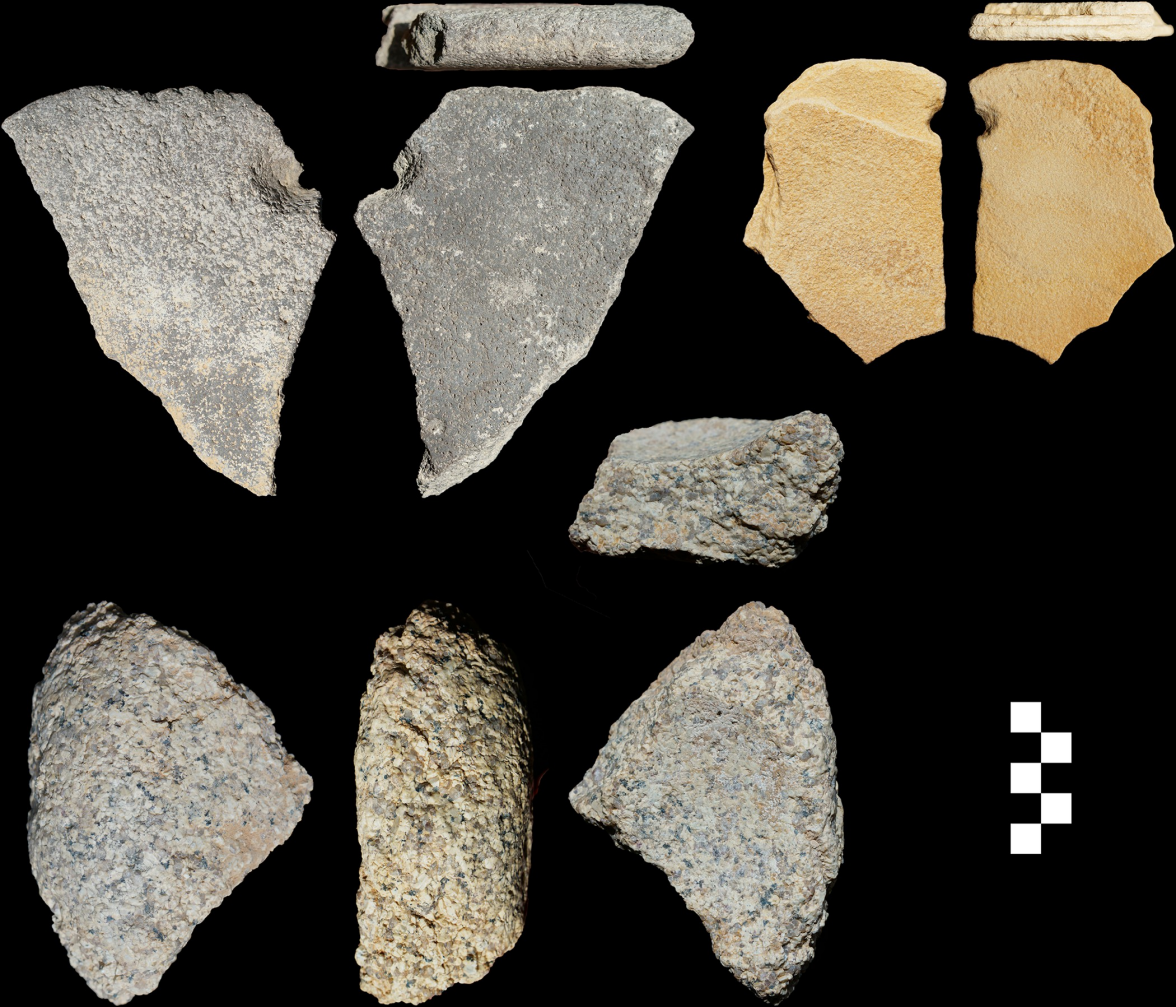
Three grinding tools from ORF2. (Top right) vesicular basalt with hole bored through; (top left) sandstone with hole bored through; (bottom) granite quern.

Four of the exotic pieces (a bottom stone and three top active stones) were manufactured using a vesicular basalt (Fig 4) which recall a raw material used for imported top active stones alongside local quartzite bottom stones at the Neolithic sites of Beidha and Jilat 7 in Jordan, with a possible source near Azraq in the east of the country [27].

**Fig 5:**
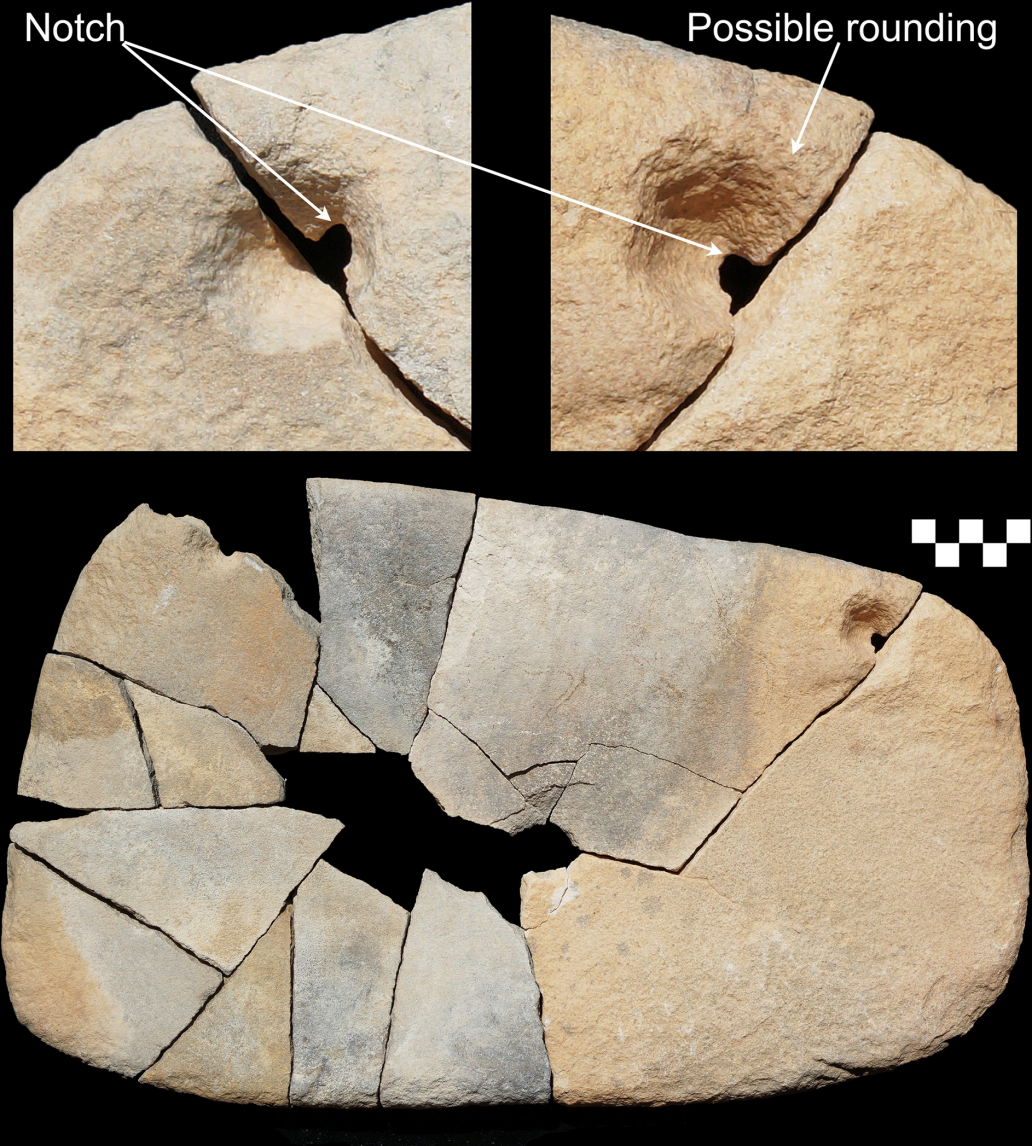
Refitting sandstone grinding stone with two holes bored through. The upper images show both surfaces of the intact hole blown up to 150% to highlight the asymmetrical notch in the perforation pointing towards the nearest edge of the tool and possible rounding wear on the adjacent side of the hole. Scale in cm.

The majority of the bottom stones show a flat morphology, with a mild concavity developing on the more heavily worn specimens. Only two dished querns occurred in the assemblage and one of these was made on an exotic granite (Fig 4 bottom). The bottom stone edges (N = 95) typically were formed from the unmodified edge of the sandstone slab on which they were made (62%), but some were shaped through unifacial and bifacial knapping (26%), some through pecking (11%) and grinding (5%), and some a combination of these. The bottom stones in general varied in degree of wear with 19% ground on both surfaces, while 12% did not appear to have been used at all. In three instances holes were bored into the bottom stones, including the exotic basalt bottom stone (Fig 4), and the most heavily used specimen that had worn through its 31 mm thickness in the centre (Fig 5). Boring holes into grinding stones has not been documented elsewhere in the Middle East to our knowledge, and we think their likely function was for attaching a strap as an adaptation to the highly mobile lifeway of the Jebel Oraf inhabitants. The holes on the near complete refitting bottom stone have an asymmetrical shape with the notch pointing towards the nearest edge of the tool and possible rounding on the adjacent side of the hole (Fig 5), as would be expected if the tool was hung with rope threaded through the holes.

The top active stones from ORF2 mostly had triangular sections for use against the heel of the hand, but two were flat. Some were modified through pecking and two may have been used as pestles as they had battering on a narrow end. Eight specimens had more than one ground surface, with one piece having five. This, coupled with the high proportion of exotic items (3/12) indicates the long use-lives of these pieces in comparison to bottom stones.

**Fig 6:**
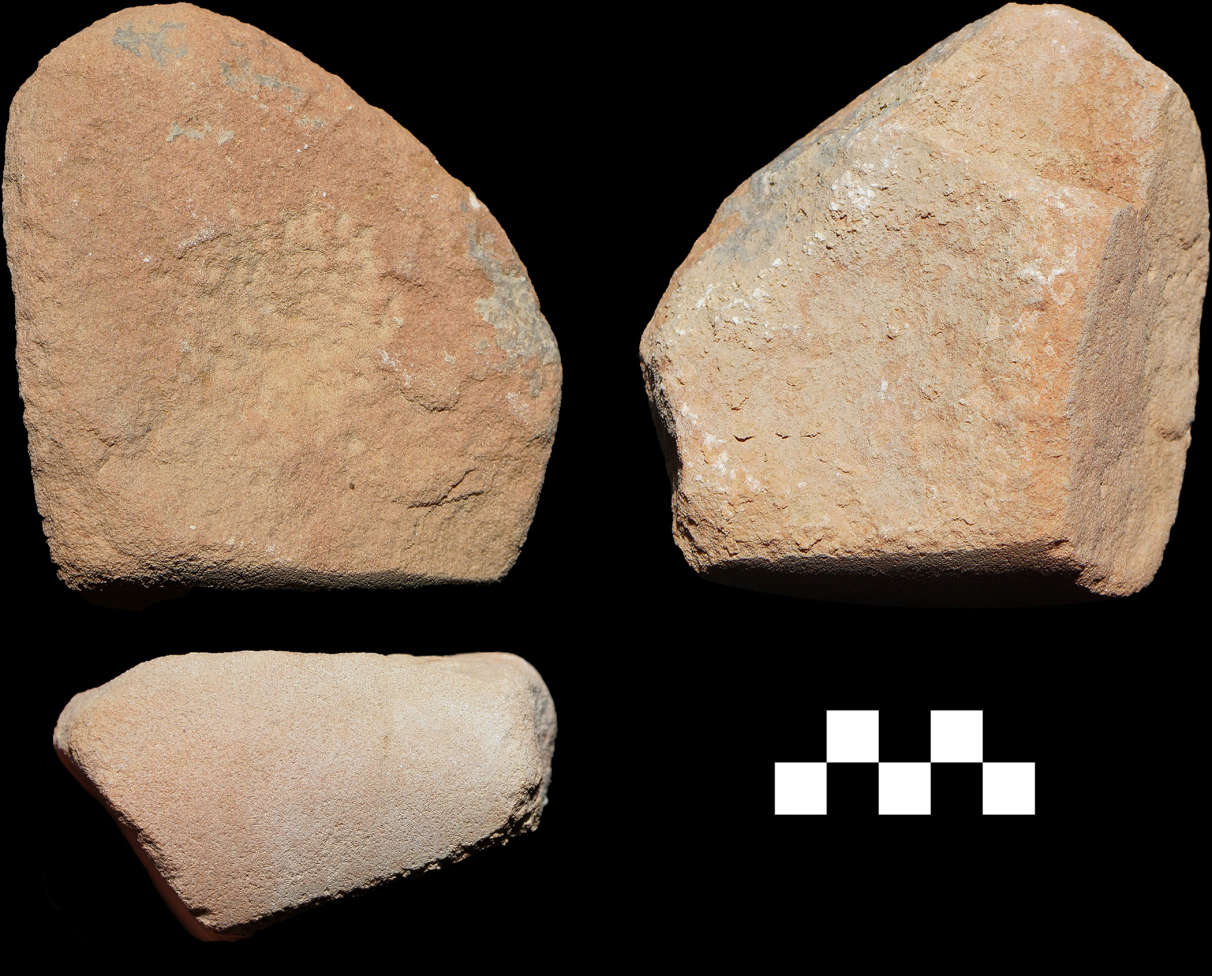
Top active stone with cupule from ORF115. Scale in cm.

The grinding tool assemblage from ORF115 was distinctive in character to ORF2. There were far fewer bottom stones (N = 11) relative to top active stones (N = 7) at the rockshelter, suggesting a functional difference between the sites. While the bottom stones were similar in form, there were no unused pieces and 27% were ground on both surfaces. The top active stones showed more extensive shaping through pecking (N = 4) and grinding (N = 1), and over half had flat grinding surfaces. There is even one piece with a cupule pecked into its surface (Fig 6). Two of the most extensively shaped top active stones were found together in the lowest occupation layer in an apparent cache. One of these has a pestle morphology, suggesting it was used for pounding as well as grinding.

From the Jebel Oraf grinding tool assemblage, five specimens were selected for the present analysis based on their recovery from buried contexts in the case of four top stones, and macroscopically visible wear and pigment in the case of the bottom stone. The four top stones were the two cached pieces and one other from ORF115, and one from ORF2 trench 10; while the bottom stone was comprised of three refitting fragments from the surface of the hearth in ORF2 trench 3.

## Methods

Functional analysis of ground stone tool assemblages has rarely been applied to sites on the Arabian Peninsula. The only exception is the work on starch residues [28, 29] at the site of Muweilah, in the Sharjah Emirate, United Arab Emirates (30, 31).

Previous work carried out in the eastern Sahara and along the Mediterranean African coast [15, 30, 31] has shown how the combined application of use-wear and plant micro-residue analysis can yield important information from grinding tools, which is not otherwise available. This type of analysis can shed light on the biography of the artefacts, the material processed with them, and, more generally, the subsistence economy of the groups which produced them.

For the plant micro-residue and use-wear analysis carried out on the artefacts studied in this paper, we followed the same protocols that were also applied to the Mid-Holocene grinding tool assemblage from the Farafra Oasis, Egypt [15], which demonstrated plant exploitation, both as characteristic wear and micro-remains, from a number of wild species. The five artefacts studied here were first sampled for plant micro-remain analysis, before washing the pieces for use-wear analysis. Samples were extracted from the examined stone tools to investigate the possible presence of starches and phytoliths, which may provide information on the use of plants in diet and/or for craft purposes. Unfortunately, no starch granules or phytoliths were retrieved from our samples.

Use-wear analysis on the archaeological tools was carried out at the Pitt-Rivers Laboratory for Archaeological Science, University of Cambridge. Before processing for use-wear analysis, the artefacts were cleaned by brushing them gently with a medium hard toothbrush and washing up liquid. The items then underwent a 60-minute sonic bath in water and washing up liquid. Use-wear analysis included both low and high magnification approaches. Low magnification observation and scanning of the selected tools’ surface micro-topography was conducted with a stereomicroscope Leica M250C at magnifications between 8x and 160x. This enables characterization of the tool’s micro-topography and detection of particular macro-wear, such as levelled areas, fractures, edge rounding, and polish, following the definitions by Adams and colleagues [32].

Subsequently, polish, and linear traces on the tool’s topography and on single quartz grains were observed and characterized through high magnification observation using a metallographic microscope Leica DM2700 at magnifications between 50x and 200x, following the approach developed by Verbaas and Tsoraki [33], as well as Hayes and colleagues [34]. The following attributes of the polish were observed and recorded: type; location and incidence; density and degree of linkage; development; reflectiveness; and directionality. Description and directionality of striations were also recorded.

The micro-wear detected on the tools’ surfaces was then compared with those present on a purpose-built experimental reference collection of sandstone artefacts, which was produced at the Institute of Heritage Science, National Research Council of Italy. In particular, these tools were used for grinding dry sorghum and abrading a dry caprine metapodial in experiments carried out for different lengths of time. For these replicas, high magnification analysis was not carried out on the actual tools, but on moulds made with a high resolution, silicon based, impression material (Provil Novo®). This product is commonly used in use-wear analysis and proved to be very reliable for recording micro-wear [35: 88]. Micro-graphs of the moulds of these experiments were taken at the Laboratory of Technological and Functional Analysis of Prehistoric Artefacts, Sapienza University of Rome, using a metallographic microscope Nikon Eclipse at magnification of 100x and 200x. The interpretations of the micro-wear on the archaeological artefacts were also based on micrographs of the actual tools which are part of the experimental reference collection of the Laboratory for Material Culture Studies at Leiden University. Finally, comparisons were also made with the experimental reference collections available in the literature [e.g. 30, 34–48].

We were cautious with the interpretation of artefacts coming from exposed contexts, which may have been subject to post-depositional agents that caused alterations of their surface, making the diagnostic use-wear features less visible [49–51]. This is the case for the majority of ground stone tools from ORF2, which were found exposed on the surface. At ORF115 Neolithic artefacts were recovered from sealed deposits (Fig 1D). Among the five sampled tools one artefact was from the surface, so was potentially affected by post-depositional agents that partially hindered micro-polish diagnostic features.

## Results of the use-wear analysis

### Grinding tool 1 –ORF115 (118)–Top active grinder/pestle

**Fig 7:**
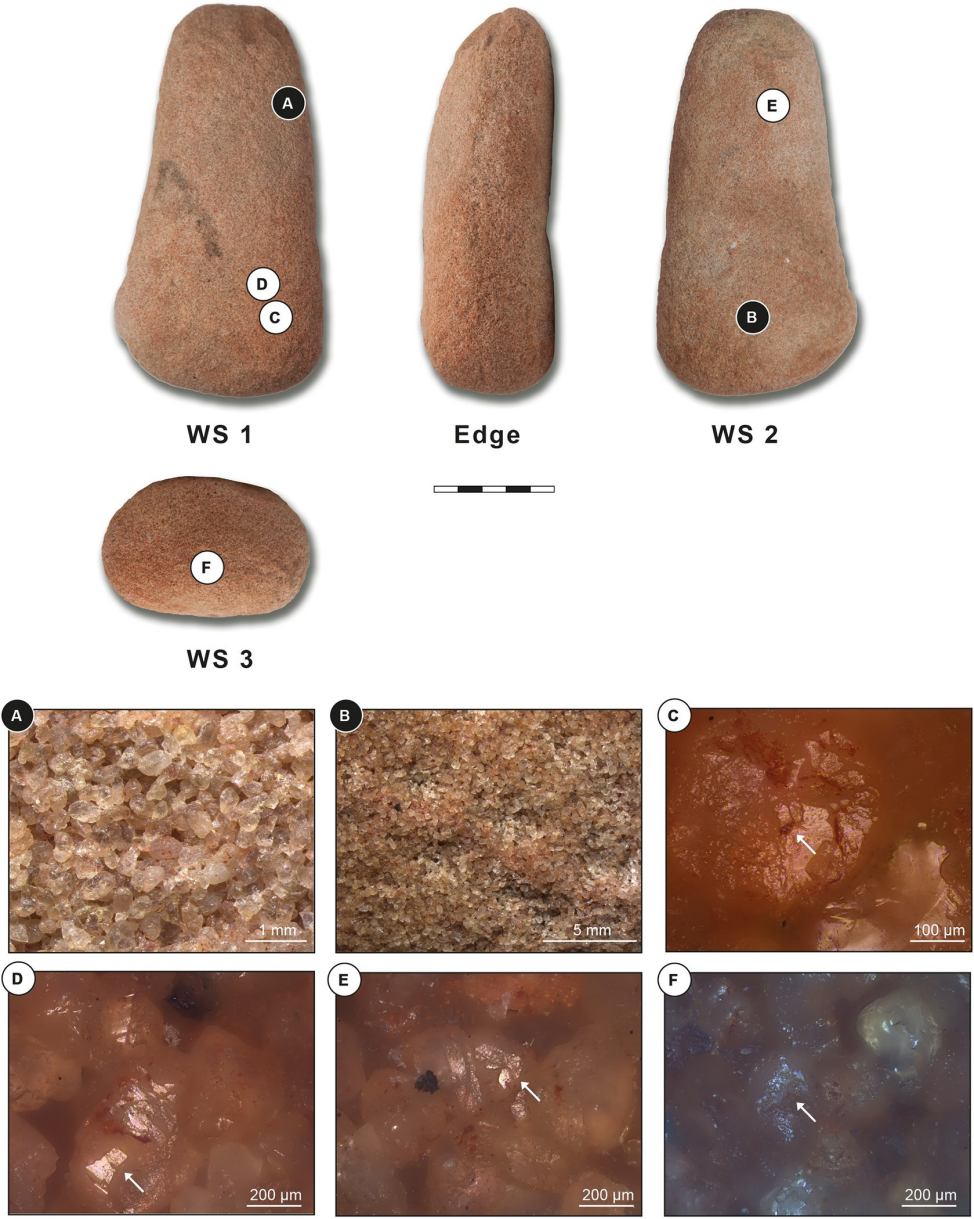
Grinding tool 1: Frustoconical pestle from ORF115, context 118. WS: working surface; White letters on black dots: Low magnification photos; Black letters on white dots: High magnification photos. (A) Loose quartz grains with abraded surfaces; (B) Grain extraction; (C) Unlinked polish in connection with micro-residues of red pigment (white arrow); (D&E) Highly reflective polish and abrasions (white arrows); (F) Moderately reflective polish associated with red pigment micro-residues (white arrow).

Grinding tool 1 is an irregularly shaped frustoconical item that has been used as a grinder/pestle. The item is made from reddish sandstone and measures 160x85x56 mm (Fig 7). The fragment has three working surfaces: a slightly convex surface (surface 1), an almost unused, slightly concave surface (surface 2), and a further convex working surface on the base of the cone (surface 3). The use intensity of the tool, as shown by microscopic observations as well as the overall morphology and number of use faces, appears low to moderate, but this may be an underestimation, due to the loose granularity of the stone leading to the repeated loss of surface material during use.

#### Grinding tool 1 –Working surface 1

At low magnification, the working surface’s topography is sinuous and irregular and shows some flattening. Micro-topography does not show heavy use-wear and the only clear signs of wear are distinct grains with abraded surfaces. These are spread all over the surface, and result from both manufacturing process and use. A possible explanation of this lack of heavier traces can be found in the raw material’s granularity which is comparatively loose. Quartz grains are attached to each other by a low presence of matrix—this would have caused a heavy extraction of quartz grains during manufacturing and use, favouring a continuous process of rejuvenation of the surface, and thus lacking heavy use-wear (Fig 7A).

At high magnification, the whole surface shows a granular, generic weak polish and micro-polish is not well developed. Some quartz grains show an unlinked polish, which is moderately to highly reflective and present on high topographies only. This type of polish, often in connection with micro-residues of red pigment, could be formed by ochre processing (Fig 7C, white arrow). Some of the quartz grains are characterized by a highly reflective polish on high topographies, and very clear abrasions, resulting from contact with mineral material (Fig 7D, white arrow).

#### Grinding tool 1 –Working surface 2

At low magnification, topography and micro-topography of working surface 2 show the same traits as surface 1, but without flattened areas. Towards surface 3 (the tool’s base and working edge) grain extractions show a clear directionality which is longitudinal and diagonal to the tool’s long axis (Fig 7B).

At high magnification, the whole working surface shows a granular, generic weak polish and micro-polish is not well developed. Equivalent to what was observed on surface 1, some quartz grains show abrasions and a high reflective polish on high topographies, which are in association with red pigment micro-residues (Fig 7E, white arrow).

#### Grinding tool 1 –Working surface 3

At low magnification, topography and micro-topography of surface 3 show the same traits as the other two. No clear wear directionality is visible.

At high magnification, the whole surface shows a granular, generic, weak polish and a high number of quartz grains show a flat, highly reflective polish on high topographies, which are likely the result of contact with mineral material. A moderately or highly reflective polish is also present on high topographies, and it is associated with red pigment micro-residues (Fig 7F). No directionality is visible.

#### Grinding tool 1 –Use interpretation

Grinder / pestle for pigment processing.

### Grinding tool 2 –ORF115 (118)–Top active grinder

**Fig 8:**
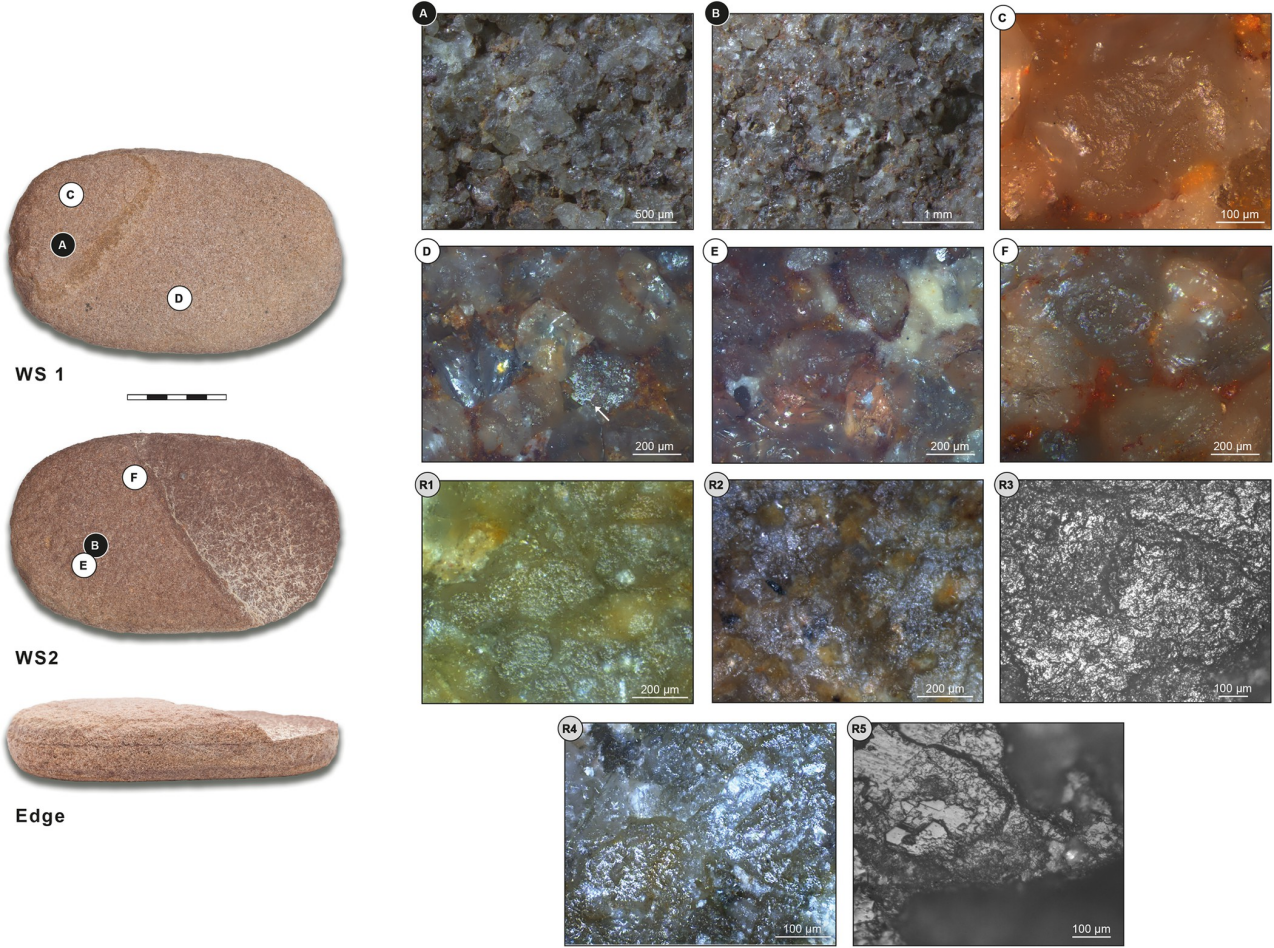
Grinding tool 2: Oval, flat top grinder from ORF115 (118). WS: working surface; White letters on black dots: Low magnification photos; Black letters on white dots: High magnification photos; Black letters/numbers on grey dots: Replica photos. (A) Abraded, contiguous, and amalgamated quartz grains; (B) Small levelled areas on high topographies; (C, E, F) Granular, moderately reflective micro-polish; (D) Smooth and moderately reflective polish (white arrow); (R1) Experimental replica used for grinding dry einkorn wheat for 600 minutes; (R2) Experimental replica used for grinding dry emmer wheat for 600 minutes; (R3) Experimental replica used for grinding dry sorghum for 360 minutes; (R4) Experimental replica used for grinding soaked bone for 180 minutes; (R5) Experimental replica used for abrading dry caprine bone metapodial for 600 minutes. (R1, R2, R4) Micrographs of the actual tools. Reference collection of the Laboratory for Material Culture Studies, Leiden University; (R3, R5) Micrographs of Provil Novo^®^ moulds. Reference collection of the Institute of Heritage Science, National Research Council of Italy, Rome.

Grinding tool 2 is an oval, flat top grinder, made from reddish sandstone, and measures 163x97x32 mm (Fig 8). The tool has two grinding surfaces: one completely preserved working surface which is flat across its length and width (surface 1), and one partly preserved working surface that is flat across the length and slightly convex across the width of the tool (surface 2). Based on both macroscopic and microscopic observations, the overall use intensity of the tool is moderate.

#### Grinding tool 2 –Working surface 1

At low magnification, the topography of working surface 1 is flat but irregular and shows some edge-rounded grains. The central area of the tool shows the lightest use-wear because it was probably the one mostly affected by rejuvenation activities such as re-pecking, traces of which are clearly visible all over the surface. The micro-topography shows edge-rounded grains are connected and concentrated around the central area. Here, some of the quartz grains also show a higher degree of wear; their surfaces are abraded, contiguous, and sometimes amalgamate (Fig 8A). Experimental comparisons, referring to dry bone abrading, are provided in the literature [37: Fig 6B, 48: Fig 2H]. Voids caused by separate grain extractions cover the whole surface; some of them, which are both superficial and deep, circular in shape, and with a U section, can be related to rejuvenation activities (e.g., pecking). Grain extractions, close to each other, are also concentrated along the two edges of the surface (deep, comet shaped, and with a U section); they show a clear directionality transversal to the long axis of the tool, which also reveals the direction in which the tool was used. The surface also shows two different types of polish: the first one is moderately reflective and visible on high and low topographies of connected grains all over the surface; the second one is also moderately reflective and visible on the high topographies of several individual grains that are spread all over the surface.

At high magnification, the surface is completely covered with a poorly granular micro-polish, moderately reflective, and in patches that are connected to each other, occurring on high topographies as well as intermediate areas (Fig 8C). The polish is more developed on high topographies which only show slight levelling. Based on the lack of distinctive micro-polish patches and topography levelling, we think this is not the result of contact with domestic cereals, but with another type of plant (possibly wild grasses?) (for comparisons see Fig 8R1-R3). Random quartz grains show a different type of polish, smooth and moderately reflective, which develops on high topographies (Fig 8D, white arrow). This does not show volume and distribution typical of contact with bone (for comparison see Fig 8R4, R5). It might be considered as one of the areas where plant micro-polish starts to link up. Considering that we detected this type of micro-feature only in few single spots, we cannot exclude the possibility that it is the result of post depositional surface modification.

#### Grinding tool 2 –Working surface 2

At low magnification, the topography of working surface 2 is flat and regular with edge rounding. The surface is characterized by a central area that shows low wear, mainly edge-rounded grains, possibly related to a first stage of exploitation of the surface. High topographies in the central area show some small, levelled areas in the very first stage of their formation (Fig 8B). The whole surface, and in particular the perimeter, show grain extraction, which are separated, both deep and superficial, circular in shape, and with a U section. There is also another type of extracted grain, which is closed and concentrated on the central area of the surface; these are comet shaped with a U section and show clear directionality, both transversal and oblique to the long axis of the tool. This confirms the direction of use already identified on working surface 1. A moderately reflective polish is developed all over the surface, on high and intermediate topographies of connected grains. The cavities are produced not only by grain extraction but also by grain fractures, which may be the result of rejuvenation activities (pecking).

At high magnification, the surface is covered by the same not well developed, granular, moderately reflective polish that is also present on working surface 1. This sometimes develops on the high topographies of single quartz grains or unlinked small patches in the central area of the surface. It can be linked to plant processing (grain grinding) (Fig 8E and 8F).

#### Grinding tool 2 –Use interpretation

Top grinder for plant processing, with possible contact of bone.

### Grinding tool 3 –ORF115 (209)–Top active grinder

**Fig 9:**
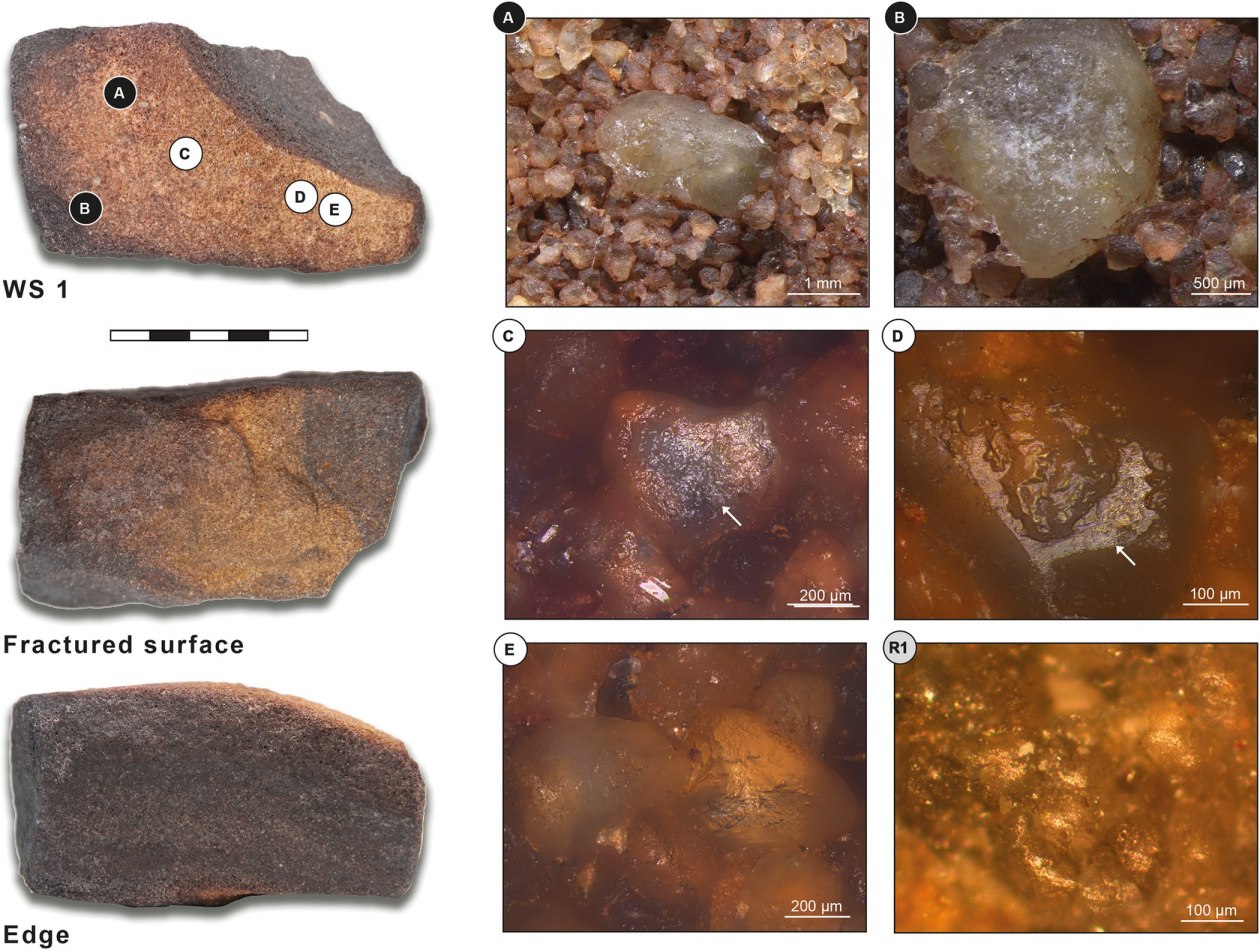
Grinding tool 3: Knapped and ground top grinder from ORF115 (209). WS: working surface; White letters on black dots: Low magnification photos; Black letters on white dots: High magnification photos; Black letters/numbers on grey dots: Replica photos. (A) Connected grain extractions; (B) Grain micro-fractures; (C) Pitted/greasy, moderately reflective polish (white arrow); (D) Possible post depositional surface modification (white arrow); (E) Generic, non-diagnostic, weak polish; (R1) Experimental replica used for grinding fresh ox bone, and contact with marrow for 60 minutes. (R1) Micrograph of the actual tool. Reference collection of the Laboratory for Material Culture Studies, Leiden University.

Grinding tool 3 is a knapped and ground, irregularly shaped top grinder, with a convex working surface. The tool has one working surface, which is convex across the length and slightly convex across the width (surface 1). The remaining five surfaces are unused and flat but irregular (Fig 9). The use intensity of this tool appears low.

#### Grinding tool 3 –Working surface 1

At low magnification, the surface topography is sinuous and irregular with edge rounding. The surface does not show substantial traces of use. As in tool 1, a possible explanation of this lack of well-developed traces could be the raw material’s granularity. Quartz grains are loosely attached by low presence of matrix, which would have resulted in a heavy extraction of quartz grains during both manufacture and use, favouring a process of rejuvenation of the surface, which thus appears little used. Micro-topography shows different types of wear: connected grain extractions are present all over the surface; they are deep, irregular in shape and show a U section (Fig 9A). Connected grain micro-fractures, both superficial and deep, are also present (Fig 9B). Polish is closed and concentrated along the curved fractured edge; it is medium reflective and developed on high topographies. The surface also shows short and shallow striations with a U section, which are present in various directions (longitudinal, transversal, and oblique), and are concentrated towards the upper area in proximity to the curved fractured edge.

At high magnification, the working surface is completely covered with a generic, non-diagnostic weak polish developed on high, intermediate, and low topographies (Fig 9E). The tool also shows other types of micro-features which are visible on a few random quartz grains all over the surface, such as a pitted/greasy, moderately reflective polish developed on high topographies (Fig 9C, white arrow). Although comparison with experimental tools (see Fig 9R1) may link this polish to contact with bone marrow, at high magnification the tool does not show the typical features which usually result from bone pounding and crushing actions for marrow extraction; for this reason we interpret the visible traces as resulting from possible bone processing. Finally, some micro-features developed on high topographies can be interpreted as a possible post depositional surface modification (Fig 9D, white arrow).

#### Grinding tool 3 –Use interpretation

Top grinder, possibly used for bone processing.

### Grinding tool 4 –ORF2 Trench 10 (152)–Top active grinder

**Fig 10:**
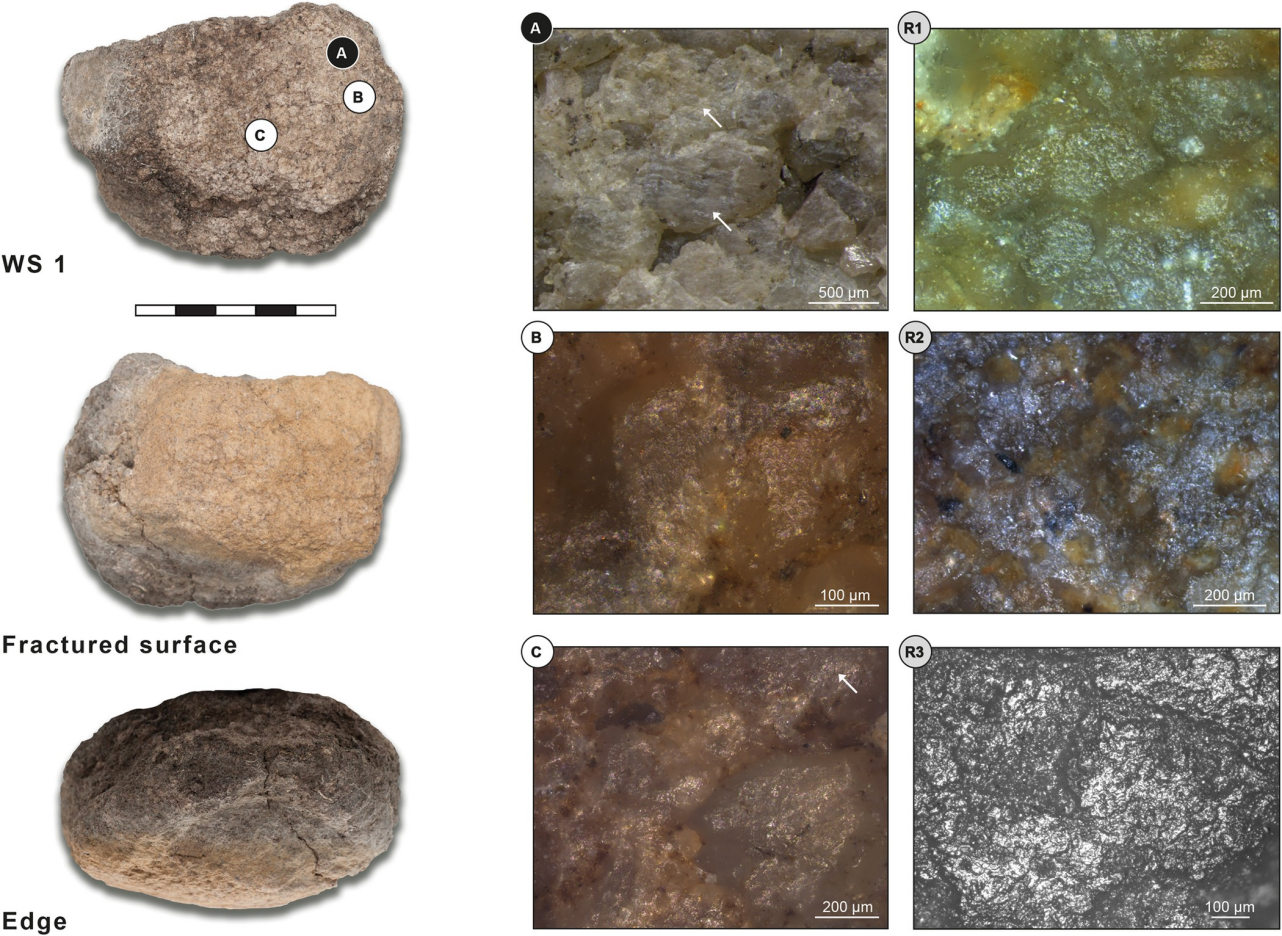
Grinding tool 4: Top grinder from ORF2 Trench 10 (152). WS: working surface; White letters on black dots: Low magnification photos; Black letters on white dots: High magnification photos; Black letters/numbers on grey dots: Replica photos. (A) Short, shallow, and parallel striations (white arrows); (B) Moderately reflective, granular polish; (C) Shallow, parallel, and narrow micro-striations (white arrow); (R1) Experimental replica used for grinding dry einkorn wheat for 600 minutes; (R2) Experimental replica used for grinding dry emmer wheat for 600 minutes; (R3) Experimental replica used for grinding dry sorghum for 360 minutes. (R1, R2) Micrographs of the actual tools. Reference collection of the Laboratory for Material Culture Studies, Leiden University; (R3) Micrograph of Provil Novo^®^ mould. Reference collection of the Institute of Heritage Science, National Research Council of Italy, Rome.

Grinding tool 4 is a top grinder of unidentifiable shape with one working surface (surface 1) that is convex across the width. The tool shows a medium use intensity (Fig 10).

#### Grinding tool 4 –Working surface 1

At low magnification, the working surface topography is flat and irregular with flattened areas (right half) and plateaus (left half). The surface can be divided into three different areas: the right part shows the most developed wear, with levelled and polished areas; the central part appears less used and shows mainly edge rounded grains, grain extractions, and fractures, while the left part could not be analysed due to the presence of a thick carbonate crust. Micro-topography shows the following features: levelled grains are sinuous and rough, developed on high and low topographies on the whole surface; on the right half they are concentrated and connected, while they are loose on the central part. A medium reflective polish is connected and mainly covers the right half of the surface, both on high and low topographies. A few short, continuous, shallow, and parallel striations with a U section can be seen along the long axis on the upper area of the surface, which is also levelled and polished (Fig 10A, white arrows). Deep irregular grain extractions are present all over the surface. They are close to each other on the central area of the surface and separated from each other on the right one. Deep connected grain fractures are also present but only on the surface’s central area.

At high magnification, the surface is covered with several connected patches of a moderately reflective granular polish, which is present on high and intermediate topographies and covers the gaps between grains. The polish is particularly developed on the surface’s right area (Fig 10B). It does not show a clear directionality, but in its central area, the surface shows some shallow, parallel, and narrow micro-striations, oblique to the tool’s long axis (Fig 10C, white arrow). This polish can be linked to plant processing, possibly grain grinding (for comparison see Fig 10R1-R3).

#### Grinding tool 4 –Use interpretation

Top grinder for plant processing (grain grinding).

### Grinding tool 5 –ORF2 (300)–Bottom grinding stone/ palette

**Fig 11:**
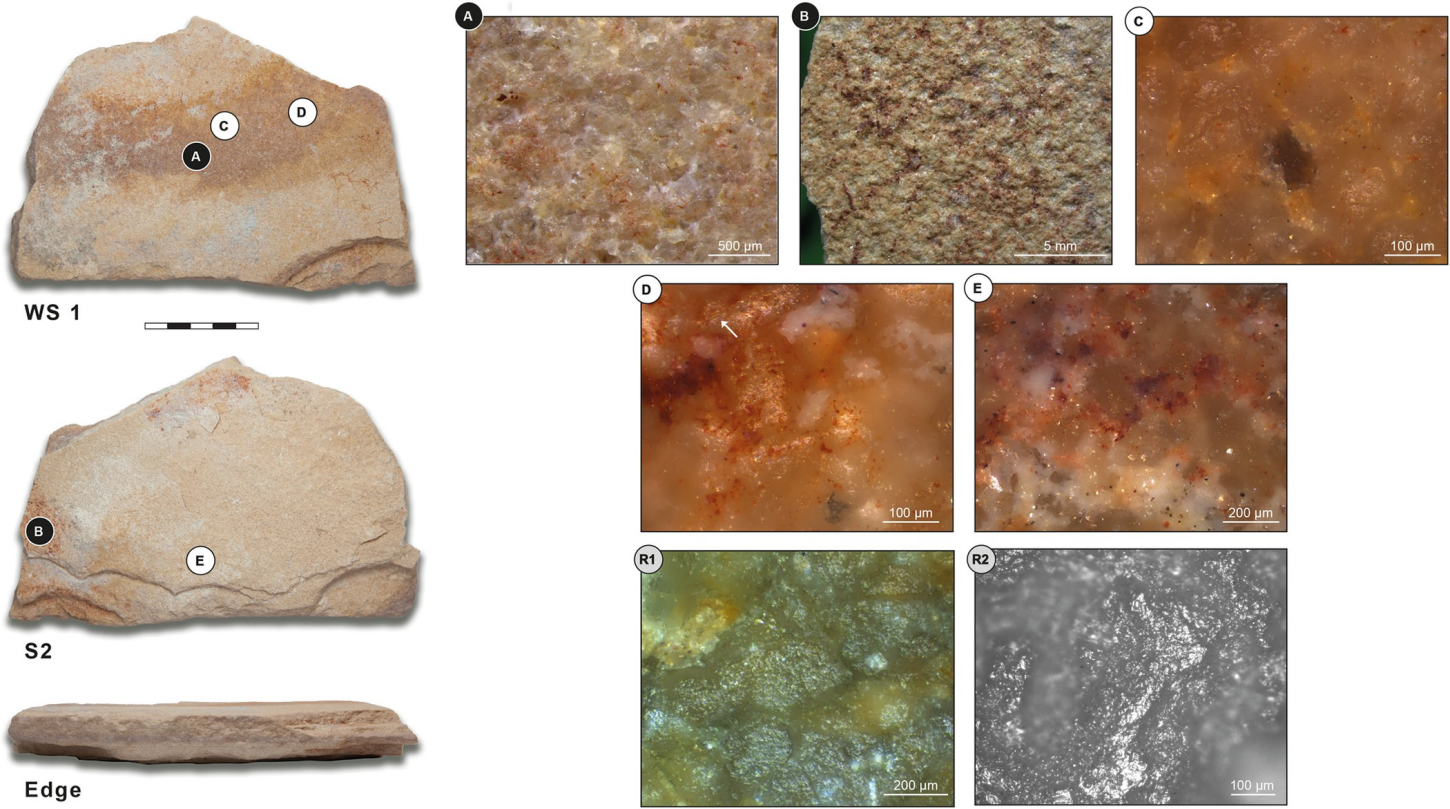
Grinding tool 5. Bottom grinding stone / Palette from ORF2 (300). WS: working surface; White letters on black dots: Low magnification photos; Black letters on white dots: High-magnification photos; Black letters/numbers on grey dots: Replica photos. (A) Grain edge rounding; (B) Residues of red pigment; (C) Granular, moderately reflective polish; (D) Granular/pitted polish with micro-striations (white arrow); (E) Generic weak polish and micro-residues of red pigment; (R1) Experimental replica used for grinding dry einkorn wheat for 600 minutes; (R2) Experimental replica used for grinding dry sorghum for 180 minutes. (R1) Micrograph of the actual tool. Reference collection of the Laboratory for Material Culture Studies, Leiden University; (R2) Micrograph of Provil Novo^®^ mould. Reference collection of the Institute of Heritage Science, National Research Council of Italy, Rome.

Fragment of a natural slab that has been knapped on the edge (Fig 11). Flake scars are visible on the lower surface, and on one end the detachments are bifacial. Tool 5 has one working surface (WS1), which is flat and slightly concave towards the fractured edge. The underlying surface has no developed wear but does have pigment residue on two of its margins (Fig 11 S2).

#### Grinding tool 5 –Working surface 1

At low magnification, the topography of the working surface is flat, regular, and shows flattening. The micro-topography shows different and clear types of use-wear: edge rounding is present all over the surface and it is connected (Fig 11A). Levelled areas are concentrated on an elongated band towards the central right area of the surface and are connected. A moderately reflective polish covers the whole surface and is present on high and low micro-topographies; a separated and loose highly reflective polish is also present and develops on high topographies only. Quartz grain extractions, possibly caused by use and not by re-pecking, due to the absence of quartz grain fractures, are separated and spread all over the surface; they are superficial, circular in shape, and show a U section. The central area of the surface shows another type of grain extraction (fine, comet-shaped, and with a U section) with a clear directionality, which is longitudinal to the long axis of the tool. The micro-topography also shows some superficial fractures, separated all over the surface. Micro-residues of red pigment are present along an elongated band, reddish in colour, which stretches between the two broken edges of the tool (Fig 11A).

At high magnification, the polish appears granular, moderately reflective, and developed in patches, which are sometimes connected to each other, or spread over large areas. It is present on high topographies and intermediate areas (Fig 11C). A highly reflective, granular/pitted polish is developed on the high topographies of single grains or small patches. In some cases, it shows directionality and micro-striations (diagonal to the long axis of the tool) (Fig 11D, white arrow). The use-wear features present on the surface can be linked to plant processing (grain grinding) (for comparison see Fig 11R1, R2). Although no clear micro-polish caused by contact with mineral material is visible on the tool, the presence of micro-residues of red pigment point to a likely reuse of the artefact as a palette.

#### Grinding tool 5 –Surface 2

Surface 2 of this fragment is unused but shows a generic weak polish and micro-residues of pigment (Fig 11B and 11E).

#### Grinding tool 5 –Use interpretation

Bottom grinding stone for plant processing (grain grinding), possibly reused as a palette for pigments.

## Discussion

The importance of grinding tools for Neolithic communities living in the Jebel Oraf basin and in the wider Jubbah Oasis is evident in their abundance. A great deal of time and effort was invested in their production: many show edge rounding, shaping and re-pecking, and some even have holes bored through them, presumably to facilitate transport (Fig 5). Despite the very temporary nature of the occupation at ORF2 and ORF115, Neolithic visitors brought grinding tools with them–some may have been brought from nearby manufacturing sites such as JKF100, while others appeared to have been carried for longer periods, until they wore thin or broke. Following their initial use, grinding stones were broken up into smaller pieces to be re-used to cover fire places. This association between hearths and grinding tools finds close parallels in the eastern Sahara, where grinding tools are frequently associated with the so-called *Steinplätze*, small hearths that are covered with small burnt stone-clusters at temporary camps [52, 53]. Perhaps surprisingly, grinding tools appear to have been closely tied to the activities and resources associated with temporary camps on the edges of palaeolakes, likely connected to the vegetation or wildlife that these localities supported.

Our results show that grinding tools were used for a range of different tasks at Jebel Oraf, including plant, pigment, and bone processing, and were subsequently broken up, re-used as fragments, then abandoned at the site. Possible secondary functions of grinding stone fragments may have been to cover the embers after the use of the hearth, or perhaps to use their surfaces for cooking or drying foods when the fire was alight. Some of the tools that showed wear associated with plant processing were later re-used as pigment palettes. The frequent use of grinding tools at ORF2, coupled with the high mobility attested in the ephemeral character of the site, may explain the perforation of some of the bottom elements to facilitate transport.

The three analysed tools from ORF115 were found in sealed contexts, directly dated to the Neolithic. Tool 1 and 2 were recovered from context 118, at the bottom of the excavated sequence and radiocarbon dated to 6000–5900 BC. Tool 3 was recovered from the edge of a hearth in context 209, in the middle of the surviving stratigraphic deposit, radiocarbon dated to 5304–5209 BC (Fig 1, Table 1). Tool 4 and 5 were both recovered from the surface of hearths at ORF2. This introduces some uncertainty in their age, particularly as most of the hearths at this site are very close to the surface, and the majority of the grinding tools were found resting on top of the hearths and thus on the modern surface (Figs 1 and 2). However, the prevalance of grinding tools in Neolithic contexts and similarities in the way they were manufactured suggests that even tools associated with later hearths were likely re-used pieces from earlier Neolithic contexts. This is particularly evident at ORF2, where grinding stone fragments that cover Neolithic hearths are still visible on the surface today and form a readily available sandstone resource at a location that is otherwise dominated by sand and lake marl. However, for surface finds such as tool 5 we cannot exclude that re-use of the tool for pigment processing occurred in later periods.

**Table 1:** List of analysed grinding tools, description and interpretation. *Probable Neolithic grinding tool fragment re-used on a Middle Islamic hearth.

Tool No	Site	Context	Tool type	Material	Weight	Dimensions	Preservation	Number of grinding surfaces	Interpretation	Associated 14C age
**1**	ORF115	118 (occupation deposit/cache)	Grinder / Pestle	Reddish sandstone	914g	160x85x56 mm	whole	3	Pigment / mineral material processing	6010–5900 calBC
**2**	ORF115	118 (occupation deposit/cache)	Top grinder	Reddish sandstone	740g	163x97x32 mm	almost intact (half of surface 2 is not preserved, but the three measurements are preserved)	2	Plant processing, possible contact with bone	6010–5900 calBC
**3**	ORF115	209 (edge of hearth)	Top grinder	Reddish sandstone	406g	90x50x51 mm	almost intact (part of the working surface is missing)	1	Possible bone processing	5304–5209 calBC
**4**	ORF2, trench 10	152 (fill of hearth); H136	Top grinder	yellow/greyish sandstone	269g	59x75x48 mm (length is not preserved)	mesial fragment	1	Plant processing	-
**5**	ORF2, trench 3	300 (surface); H63	Bottom grinding stone / Palette	yellow sandstone	649g	170x109x22 mm (length and width not preserved)	1 of 3 fragments belonging to the same tool	2	Plant processing, reused as a palette for pigments	1306–1411 CE*

### Plant processing

Excavations at ORF2 and ORF115 did not reveal macro-botanical remains, despite extensive sieving, presumably due to poor preservation. Use-wear analysis now confirms plant processing in at least three of the five analysed grinding tools (tools 2, 4 and 5) at these sites. Although we cannot currently say exactly what plants were processed, wild grasses seem likely based on comparative samples. The time and technological investment in grinding plants highlights their importance in the Neolithic economy of northern Arabia, with plants likely ground, cooked, and consumed at the site.

There is currently no evidence for the use of domesticated grains in the Neolithic of northern Arabia. However, processing of wild grains and tubers are well attested at the Natufian site of Shubayqa 1 in eastern Jordan, where they were ground into flour and baked into bread-like products [54]. Exploitation of similar grinding tools for processing wild grasses (especially sorghum) was also evidenced in contemporary and environmentally similar contexts of the eastern Sahara oases [15, 20, 26]. Processing of wild grains and tubers, processing of domesticated grains and other plant foods, as well as processing of plant fibres for non-alimentary purposes such as basket or rope production and other crafts therefore remain possibilities at Jebel Oraf. Given the layout of the more substantial hearths found at ORF2, which are lined with large stones, the making of bread-type foods is plausible, especially if these could be used as easily transportable foodstuffs (as suggested for Natufian hunter-gatherers in eastern Jordan by Arranz-Otaegui and colleagues [54]).

### Bone processing

Evidence from faunal remains shows that burning of bones was directly associated with the use of hearths [1] and that meat was cooked and consumed at the site. Identified species include both wild and domestic species: cattle, gazelle, sheep/goat, and oryx, as well as ostrich. Fragmentation was high, which was attributed to poor preservation [1]. Use-wear analysis indicates that grinding tools were used for the processing of animal materials and bone. Some of the bone fragmentation observed at ORF2 and ORF115 may have been caused by the breaking of bones to access bone marrow. Bone marrow has been shown to have been a reliable source of fat in the early Holocene of the southern Levant [55] and this may also have been the case at Jebel Oraf [1]. The extraction of bone marrow requires more effort than meat, but less effort than the extraction of fat [56: 142], and marrow may have been a valuable source of food in the marginal environment of the Neolithic in Jubbah. Although breaking or crushing of bone was not confirmed by use-wear analysis, evidence for bone processing at Jebel Oraf might relate to marrow extraction, as a further facet in the diverse exploitation of animal resources in an environment where successive wet phases and droughts [57] may have required Neolithic hunter-herders to adapt to periods of abundance and scarcity.

### Pigment processing

**Fig 12:**
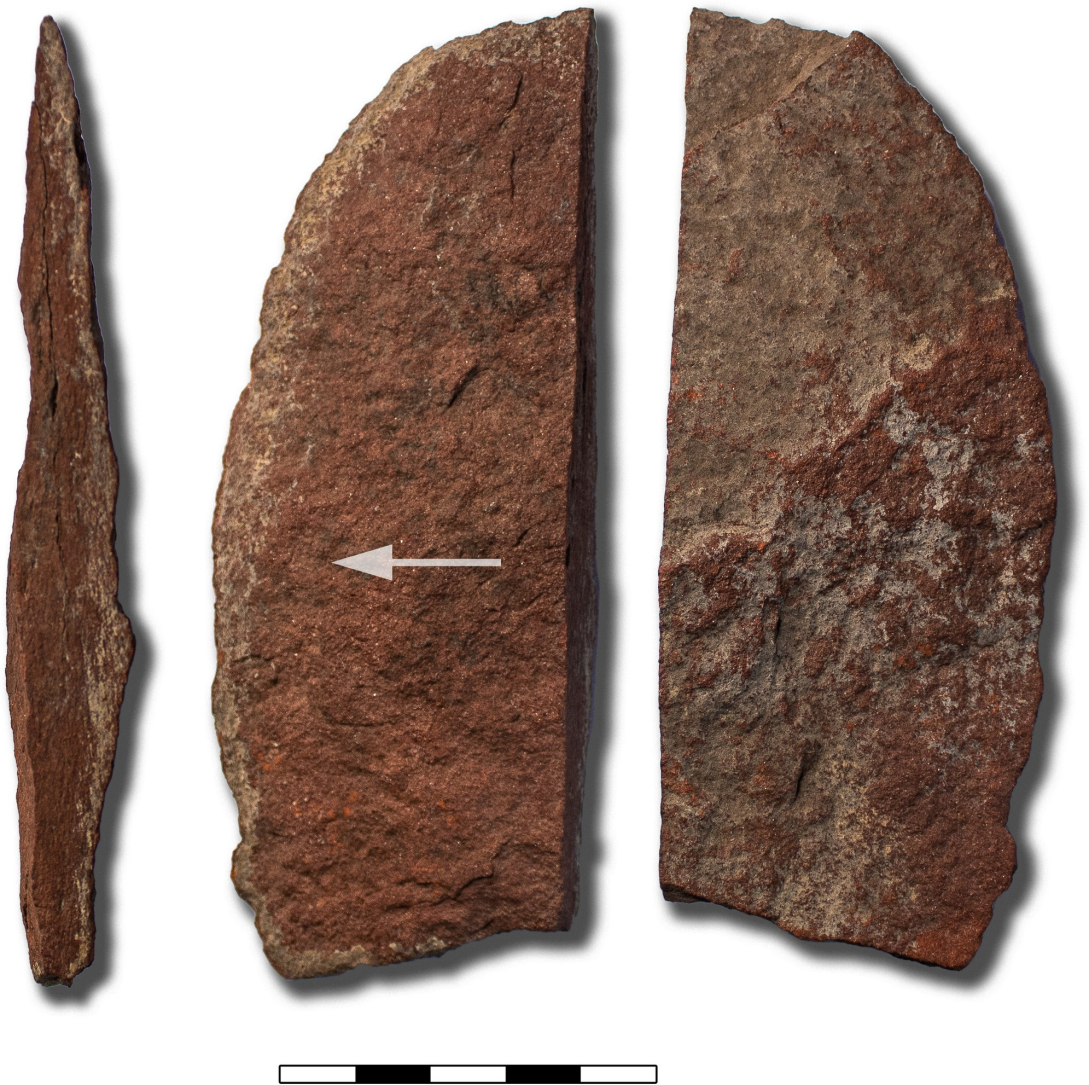
Shale flake from ORF2 associated with a Neolithic hearth (hearth 88). From left to right: the platform (probably the originally exposed surface of the shale seam), the ventral surface (with an arrow indicating the detachment force direction), and the dorsal surface. Scale in cm.

**Fig 13:**
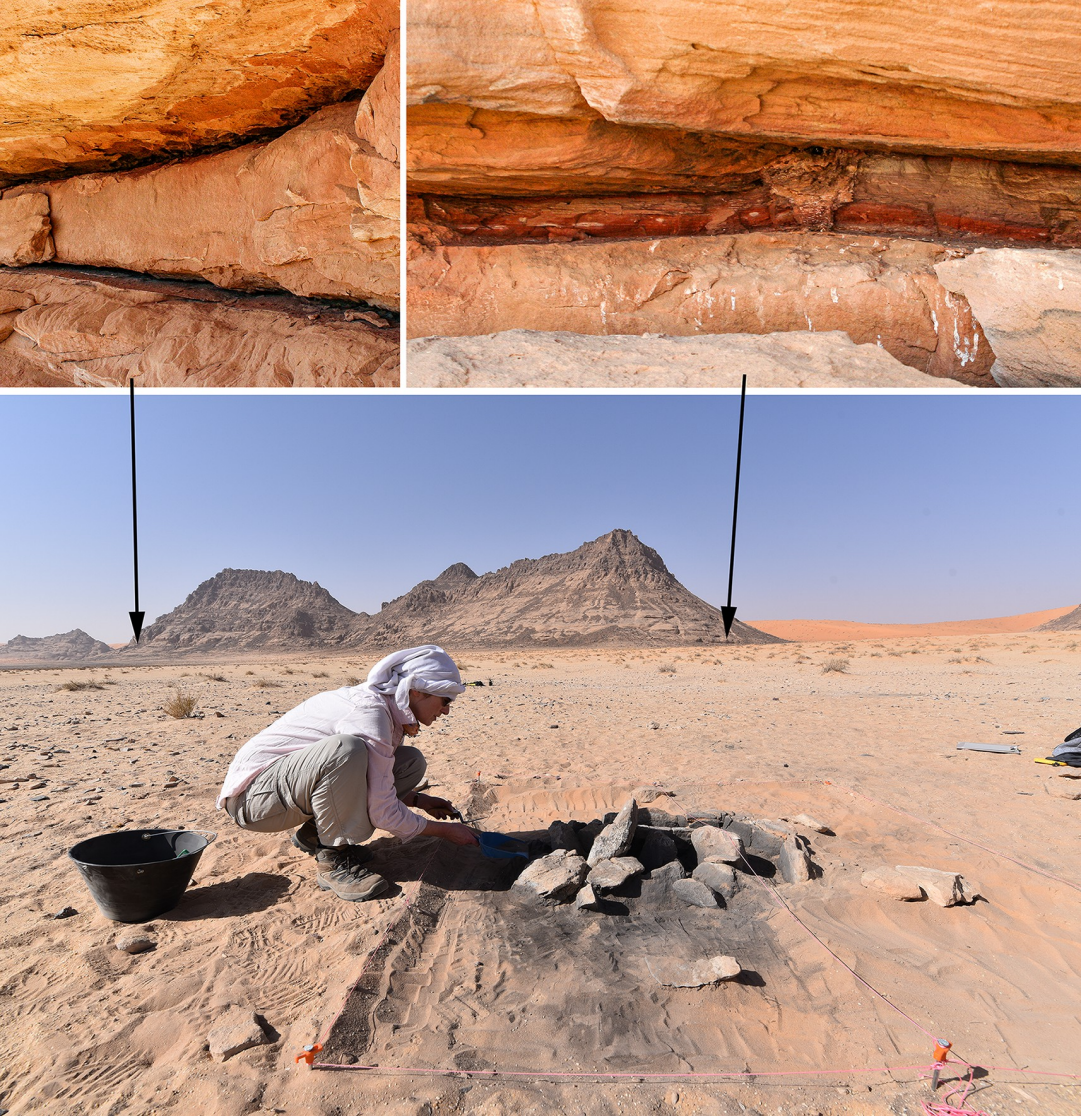
Shale sources at Jebel Oraf. (Bottom) Excavation of hearth 145, Trench 4 at ORF2. Jebel Oraf is visible in the background; shale sources are marked with arrows. (Top) photographs of shale seams identified at Jebel Oraf.

The Neolithic rock art of northern Arabia is iconic and thousands of engravings depicting hunting scenes and cattle herds have been documented in recent years [12, 58]. However, despite this abundance, evidence for the use of pigment in Neolithic art has so far been absent, with the exception of a single painted rock that formed part of a mustatil [11], and a small group of paintings at Jebel Qattar [59]. Conversely, the use of pigment in rock art is well attested for the Iron Age and historic periods [60]. In our analyses pigment processing was evident in two of the five samples as one of the activities carried out by the local Neolithic groups. Moreover, two larger pieces of red shale were documented during excavations at ORF2, one of which was a large flake (Fig 12). This piece was associated with hearth 88, which was radiocarbon dated to 5293–5068 BC [1]. Multiple sources of this red shale were observed at the base of at least two jebels in the Jubbah Oasis. At Jebel Oraf two bands of red shale appear to stretch across the sandstone and in some locations, there is evidence of past extraction (Fig 13). Our results now show that red shale has been processed in Jubbah since at least the Neolithic period. One function of the pigment was its use in rock paintings. A small group of painted cattle at Jebel Qattar, approximately 28 km to the northeast, on the eastern end of the Jubbah palaeolake basin can be attributed to the Neolithic period based on their content and stylistic criteria [59], with our results further supporting this assessment.

**Fig 14:**
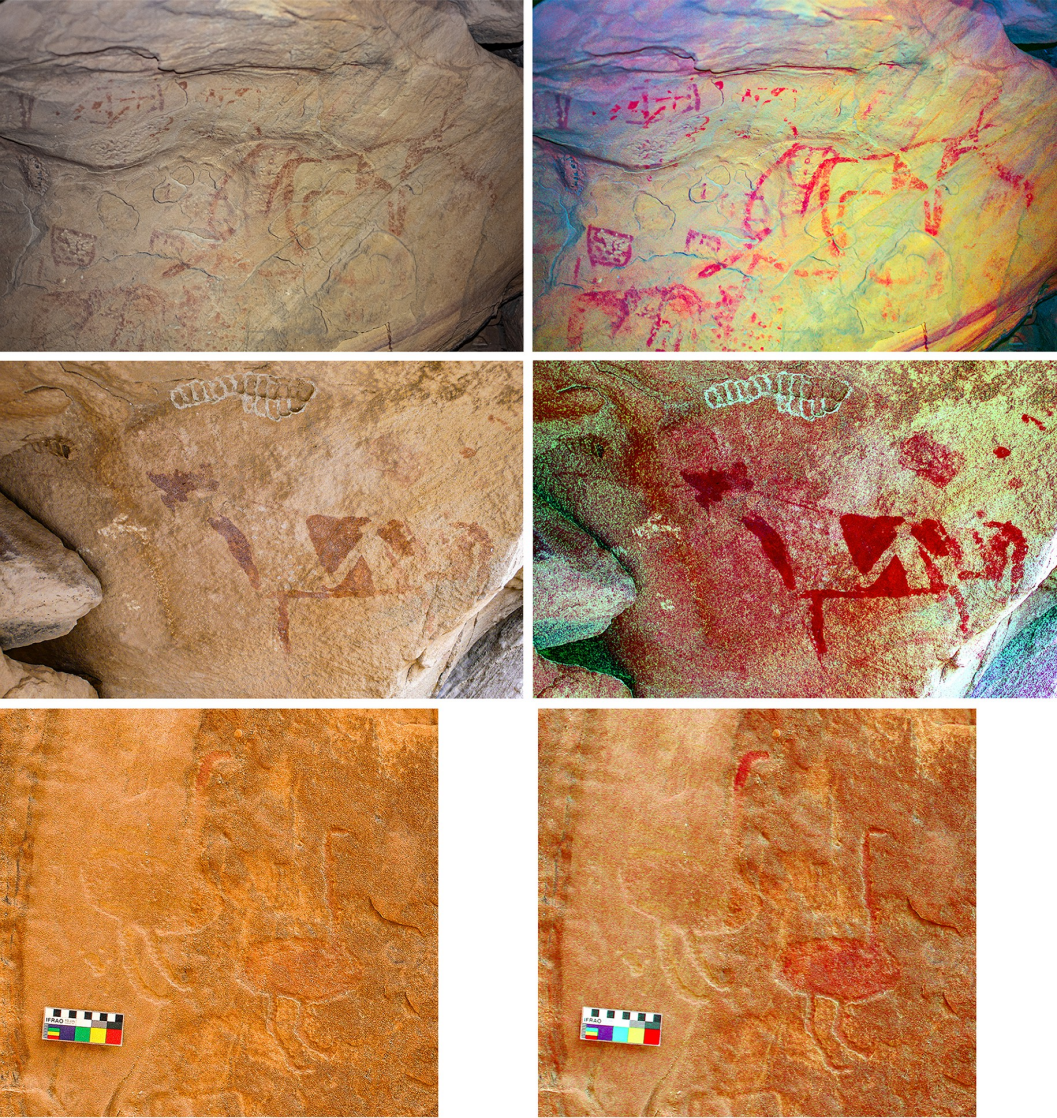
Painted rock art in the wider Jubbah Oasis. (Top left) Panel with two cattle at Jebel Qattar. (Top right) Photo enhanced with DStretch software, lds setting. (Middle left) Painted cattle with triangular decoration at Jebel Qattar. (Middle right) Photo enhanced with DStretch, yrd setting. (Bottom left) Two engraved and painted ostriches from Jebel Oraf. (Bottom right) Photo enhanced with DStretch, yrd setting.

Some painted panels show the use of at least two different shades of red. For example, Fig 14 (top) shows some geometric, rectangular shapes in a darker red, and two cattle in a lighter, more orangey red. Both shades of red are also visible in the shale seams that were identified at Jebel Oraf (Fig 13, top right), with similar shale sources also observed at other Jebels in the Jubbah Oasis. Painted rock art was also recorded at Jebel Oraf, where several panels of engraved and then painted images of uncertain age were recorded (Fig 14, bottom). Our results show that the widely available red shale was exploited for pigment production more commonly in the Neolithic than previously known and continued over several millennia. It is therefore possible that originally more Neolithic paintings existed, which have now been lost to erosion. Notably, Neolithic painted art is currently only visible on the most sheltered rock art panels in the Jubbah basin and on a single painted rock that formed part of a mustatil in the southern Nefud Desert [11]. However, the processing and use of pigment for other purposes, for example to colour materials or for cosmetics also remain a possibility.

## Conclusion

The results from our use-wear analyses confirm that grinding tools at Jebel Oraf were used for a range of purposes: processing plants, bone, and pigment. The association of the analysed tools with the Neolithic allows for insights into the subsistence acvitities of this period. Three of the five tools showed evidence of bone processing. Fragmentation of bones observed at ORF2 and ORF115 suggests that ground tools may have been used to break open bones and access the marrow, perhaps an important source of nutrition that could be stored for several weeks to provision long journeys across the Nefud [see for example 56, 61]. On three tools use-wear analysis identified evidence for plant processing. The apparently common use of grinding tools for plant processing and the overall abundance of grinding tools at ORF (with 162 recorded at ORF2 alone) suggests plants and plant foods were economically important for Neolithic people who have previously been characterized as hunter-herders. The production of bread-type foods, whether from wild or domesticated plant sources, and the use of plant fibres in crafts such as basketry and rope making would accord well with a highly mobile lifestyle requiring transportable foodstuffs.

Two of the analysed tools showed evidence for pigment processing (tool 1 and 5), including one tool from a securely dated and sealed Neolithic context. This provides a crucial link to rock art production in the area, which includes some painted Neolithic panels of domesticated cattle. The discovery of red shale pieces in Neolithic contexts suggests that painted rock art may have been more common in the Neolithic of northern Arabia than its surviving distribution.

Our research has shown that the application of use-wear analysis to grinding tools can yield significant new information that is otherwise unavailable. This type of analysis has only rarely been applied to archaeological materials from the Arabian peninsula, but can inform us on the manufacture, use, and re-use of objects, which in turn provides insight into the subsistence, economy, and art of the people who produced them.
